# Retraction: Measles-mumps-rubella vaccination timing and autism among young African American boys: a reanalysis of CDC data

**DOI:** 10.1186/2047-9158-3-22

**Published:** 2014-10-03

**Authors:** Brian S Hooker

**Affiliations:** 1 Simpson University, Redding, CA, USA

## Retraction

The Editor and Publisher regretfully retract the article [1] as there were undeclared competing interests on the part of the author which compromised the peer review process. Furthermore, post-publication peer review raised concerns about the validity of the methods and statistical analysis, therefore the Editors no longer have confidence in the soundness of the findings. We apologise to all affected parties for the inconvenience caused.
